# Lignin Composition and Structure Differs between Xylem, Phloem and Phellem in *Quercus suber* L.

**DOI:** 10.3389/fpls.2016.01612

**Published:** 2016-10-27

**Authors:** Ana Lourenço, Jorge Rencoret, Catarina Chemetova, Jorge Gominho, Ana Gutiérrez, José C. del Río, Helena Pereira

**Affiliations:** ^1^Centro de Estudos Florestais, Instituto Superior de Agronomia, Universidade de LisboaLisboa, Portugal; ^2^Consejo Superior de Investigaciones Científicas, Instituto de Recursos Naturales y Agrobiología de SevillaSeville, Spain

**Keywords:** cork, DFRC, milled lignin, NMR, phloem, Py-GC/MS, *Quercus suber*, xylem

## Abstract

The composition and structure of lignin in different tissues—phellem (cork), phloem and xylem (wood)—of *Quercus suber* was studied. Whole cell walls and their respective isolated milled lignins were analyzed by pyrolysis coupled with gas chromatography/mass spectrometry (Py-GC/MS), two-dimensional nuclear magnetic resonance spectroscopy (2D-NMR) and derivatization followed by reductive cleavage (DFRC). Different tissues presented varied *p*-hydroxyphenyl:guaiacyl:syringyl (H:G:S) lignin compositions. Whereas lignin from cork has a G-rich lignin (H:G:S molar ratio 2:85:13), lignin from phloem presents more S-units (H:G:S molar ratio of 1:58:41) and lignin from xylem is slightly enriched in S-lignin (H:G:S molar ratio 1:45:55). These differences were reflected in the relative abundances of the different interunit linkages. Alkyl-aryl ethers (β–*O*–4′) were predominant, increasing from 68% in cork, to 71% in phloem and 77% in xylem, as consequence of the enrichment in S-lignin units. Cork lignin was enriched in condensed structures such as phenylcoumarans (β-5′, 20%), dibenzodioxocins (5–5′, 5%), as corresponds to a lignin enriched in G-units. In comparison, lignin from phloem and xylem presented lower levels of condensed linkages. The lignin from cork was highly acetylated at the γ-OH of the side-chain (48% lignin acetylation), predominantly over G-units; while the lignins from phloem and xylem were barely acetylated and this occurred mainly over S-units. These results are a first time overview of the lignin structure in xylem, phloem (generated by cambium), and in cork (generated by phellogen), in agreement with literature that reports that lignin biosynthesis is flexible and cell specific.

## Introduction

Lignin is the second most abundant polymer in vascular plants. Lignin deposition in the cell wall has a major importance for plant physiology and development: (i) by acting as the mechanical support of plant organs, it allows an upright growth and large sizes; (ii) it provides strength and rigidity to the cells; (iii) it allows transport of water and solutes in the vascular system due to its hydrophobicity and mechanical resistance; and (iv) it is associated to protection against pathogens (Boudet, 2000; Donaldson, 2001; Boerjan et al., 2003; Vanholme et al., 2008).

Revealing the lignin structure and the lignification mechanism has been the subject of extensive research along the years. It has been well established that lignin is synthesized from the combinatorial oxidative coupling of three main *p*-hydroxycinnamyl alcohol monomers (*p*-coumaryl, coniferyl, and sinapyl alcohols) and related compounds (Boerjan et al., 2003; Ralph et al., 2004a; Vanholme et al., 2010). In the last decade other monomers have been recognized as participating in lignin polymerization, including hydroxycinnamic acids and aldehydes, as well as coniferyl and sinapyl acetates or coumarates (e.g., Ralph et al., 2004a; Grabber et al., 2010; Ralph, 2010). After their synthesis, the lignin monomers are transported to the cell wall where they are polymerized in a combinatorial fashion by free-radical coupling mechanisms in a reaction mediated by peroxidases, generating a variety of structures within the lignin polymer (Boerjan et al., 2003; Ralph et al., 2004a). The relative proportion of lignin monomers varies between plants and changes depending on the tissue, cell location or environmental conditions. The lignin molecule has a high chemical flexibility, i.e., the plant produces a lignin with a specific composition depending on the precursors that are been deposited in the lignifying zone (Boudet, 2000).

The composition and structural characteristics of the lignin have been studied in different plant tissues, including wood xylems, triggered by the importance of wood delignification for the pulp industry (Tsutsumi et al., 1995; Rencoret et al., 2008; Santos et al., 2011; Lourenço et al., 2013), and also in herbaceous plants (del Río et al., 2007a, 2012a,b; Buranov and Mazza, 2008; Marques et al., 2010). The content and composition of lignins vary among taxa, cell types, and individual cell-wall layers, and also with environmental conditions or plant growth stage (Ralph and Hatfield, 1991; Buranov and Mazza, 2008; Rencoret et al., 2008). It has become evident that lignin formation and composition is cell specific, e.g., differing between tracheary elements, sclerenchyma cells and endodermal cells, and presenting also a distinctive feature at sub-cellular localization (Barros et al., 2015). For instance, the cell walls of xylem vessels have a predominance of H-units and cell corners and middle lamella present a G-lignin, while the cell wall of fibers is rich in S-units (Schuetz et al., 2013).

The structure of the lignin in barks is much less known, and only few studies exist on comparative lignin composition of xylem and bark of the same species, such as in *Tectona grandis* (Lourenço et al., 2015) and *Pinus sylvestris* (Normark et al., 2014). The fact that bark is a heterogeneous material including phloem, periderm, and eventually rhytidome, which have different biological origin, adds to the complexity; phloem and xylem cells result from the meristematic activity of cambium, while the periderm originates from the activity of phellogen that forms a thin layer of phelloderm cells to the inside and phellem (cork) cells to the outside (Esau, 1960).

The comparative analysis of lignin composition and structure in different tissues i.e., xylem, phloem, and periderm, of the same species has not been reported so far. An interesting case of study is the cork oak (*Quercus suber*), where the periderm forms a thick layer of phellem that is now the source of commercial cork (Pereira, 2007). The cork tissues are homogeneous as regards their cell type structure and are chemically out-singled by the presence of suberin as the major structural cell wall component (Pereira et al., 1987; Pereira, 1988, 2013; Conde et al., 1998). Lignin is the second most important component of cork cell walls, and, together with suberin, contributes decisively to cork properties e.g. elasticity and resilience (Pereira, 2015). Previous studies of cork lignin from different species, including *Q. suber, Q. cerris, Betula pendula* and *Pseudotsuga menziesii* (Marques et al., 1994, 1996, 1999, 2006, 2015; Marques and Pereira, 2013), revealed that its composition was quite different to that from xylem lignin (Marques and Pereira, 2014).

In this context, the aim of this work is to study the differences in composition and structure of the lignins from three tissues—xylem, phloem and phellem—of *Q. suber*. For this, the milled lignins (ML) were isolated according to the classical Björkman procedure (Björkman, 1956) and analyzed by the use of an array of analytical techniques, including analytical pyrolysis, 2D-nuclear magnetic resonance spectroscopy (2D-NMR), and derivatization followed by reductive cleavage (DFRC). The results provide a first time overview of the differences in the lignin structure in the xylem and phloem tissues originating from the vascular cambium, and in the cork generated by the phellogen.

## Materials and methods

### Samples

*Quercus suber* L. samples were taken from a 6-year-old tree from discs taken between 1.0 and 1.3 m of stem height. The xylem, phloem and cork tissues were manually separated from each other using a chisel. Each material was milled in a knife mill (Retsch SM 2000), passing through a 6 × 6 mm sieve, and sieved in a Retsch AS 200. One sample was taken from the 40–60 mesh fraction (250–425 μm) for chemical analysis. A mixture of all the granulometric fractions was successively extracted with dichloromethane, ethanol and water for 24 h each. The extracted samples were oven-dried at 60°C, and milled in a knife mill (IKA MF10) passing through a 100-mesh (<180 μm) sieve to obtain a fine granulate for lignin isolation.

### Chemical analysis

Two aliquot samples from the 40–60 mesh fraction (250–425 μm) from cork, phloem and xylem were chemically characterized, following procedures adapted from TAPPI standard methods (TAPPI, 2004): ash content (TAPPI T211 om-02), total extractives determined from successive Soxhlet extraction with dichloromethane, ethanol and water (TAPPI T204 cm-07), total lignin determined as the sum of Klason lignin (TAPPI T222 om-11) and acid-soluble lignin (UM 205 om-83). Neutral monosaccharide composition was determined in the hydrolysate from the lignin analysis. The monosaccharides were separated by High Pressure Ion Chromatography using a Dionex ICS-3000 system equipped with an electrochemical detector; the mobile phase was NaOH (2 mM solution) with a flux of 1.0 mL/min at 25°C; and the column used was Aminotrap plus Carbopac SA10. The results were reported as percent of initial material. In the case of cork, the suberin content was determined in the extractive-free material by methanolysis, as described in Pereira (2013), and the lignin was determined using the suberin-free material (according to TAPPI T222 om-11).

### Anatomical observation

A sample of each tissue was impregnated with DP1500 polyethylene glycol, and transverse microscopic sections of approximately 17 μm thickness were cut with a microtome (Leica SM 2400). The cork and phloem sections were stained with triple staining astra blue/crysoidine/sudan IV, and the xylem sections with safranin. All the sections were observed in a light microscopic using Leica DM LA and photomicrographs were taken with a Nikon Microphot-FXA.

### Lignin isolation

Milled lignins from xylem, phloem and cork were isolated according to a procedure adapted from Björkman (1956). The granulates were finely ball-milled using a 500 mL agate jar and agate ball bearings (20 × 20 mm) in a Retsch PM100 planetarium ball mill, at 400 rpm, during 5 h with 5 min breaks after every 5 min of milling. The ball-milled powder was extracted with dioxane-water (96:4, v/v) using 25 mL of solvent g^−1^ of sample under agitation for 12 h. The solution was centrifuged, and the supernatant evaporated to dryness at 40°C at reduced pressure. The residue, called raw milled lignin (raw ML) was dissolved into a solution of acetic acid:water (9:1, v/v) using 20 mL of solvent g^−1^ of raw ML. The lignin was precipitated into stirred cold water (225 mL g^−1^ of raw ML), the precipitate was centrifuged, dried and milled in an agate mortar. This residue was dissolved in a 1,2-dichloroethane:ethanol solution (2:1, v/v) using 25 mL of solvent/g of lignin. After centrifugation to remove undissolved matter, the lignin in the supernatant was precipitated by adding the solution drop wise into diethyl ether, and the obtained residue was separated by centrifugation. The solid residue was suspended in diethyl ether overnight, centrifuged, and finally suspended in petroleum ether overnight. The final purified milled lignin was recovered by centrifugation and dried under N_2_ flow. The final yields ranged from 15 to 20% based on the Klason lignin content.

### Analytical pyrolysis (Py-GC/MS)

The milled lignins (1.7 mg) were pyrolysed in a EGA/Py-3030D micro-furnace pyrolyzer (Frontier Laboratories Ldt.), connected to an Agilent 7820A GC system equipped with a DB-1701 fused-silica capillary column (60 m × 0.25 mm i.d. × 0.25 μm film thickness), and to a Agilent 5975 Mass detector (EI at 70 eV). The pyrolysis was performed at 500°C (1 min). The oven temperature was programmed from 45°C (4 min) to 280°C at a heating rate of 4°C min^−1^, and held at 280°C during 10 min. The GC/MS interface was kept at 280°C and the injector at 250°C. The carrier gas was Helium with a flow of 2 mL min^−1^. The compounds were identified using the literature (Faix et al., 1990; Ralph and Hatfield, 1991) and the Wiley and NIST libraries. Peak molar areas were calculated for each compound (by dividing the peak area by the respective molecular weights), the summed molar areas were normalized and the data expressed as percentage.

### 2D-NMR spectroscopy

Around 100 mg of the whole cell wall (CW) material and 30 mg of the isolated milled lignins were dissolved in 1 mL and 0.75 mL of DMSO-*d*_6_, respectively, for the NMR analysis. HSQC (heteronuclear single quantum correlation) spectra were recorded at 300 K on a Bruker AVANCE III 500 MHz spectrometer (Bruker Biospin, Fallanden, Switzerland), equipped with a cryogenically cooled 5 mm TCI gradient probe with inverse geometry (proton coils closest to the sample). The 2D ^13^C-^1^H correlation spectra were obtained using an adiabatic HSQC pulse program (Bruker standard pulse sequence “hsqcetgpsisp2.2”). The spectral widths were from 10 to 0 ppm (5000 Hz) in F_2_ for ^1^H dimension, with an acquisition time of 100 ms (CW) or 145 ms (ML), and a recycle delay (d1) of 1 s. For the ^13^C dimension, the spectral width was from 200 to 0 ppm (25,168 Hz) in F_1_, being collected 256 increments of 32 scans for a total acquisition time of 2 h 34 min (CW) and 2 h 40 min (ML). The ^1^*J*_CH_ used was 145 Hz. Processing used typical matched Gaussian apodization in ^1^H and a squared cosine bell in ^13^C. The central solvent peak was used as an internal reference (δ_C_ 39.5; δ_H_ 2.49 ppm). 2D NMR HSQC cross-signals were assigned after comparison with data from literature (Ralph et al., 1999, 2004b; Capanema et al., 2005; Rencoret et al., 2011; del Río et al., 2012a,b). A semiquantitative analysis of the volume integrals of the HSQC correlation peaks was performed using Bruker's Topspin 3.1 processing software. The relative abundances of side-chains involved in the different inter-unit linkages were estimated in the aliphatic oxygenated region from the C_α_–H_α_ correlations, except for α-oxidized β−*O*−4 substructures (Aox) and cinnamyl alcohol end-groups (structure **I**, **Figure 5**), for which C_β_–H_β_ and C_γ_–H_γ_ correlations were used. In the aromatic/unsaturated region, C_2_–H_2_ correlations from H, G and S lignin units and from ferulates were used to estimate their relative abundances.

### Derivatization followed by reductive cleavage method modified (DFRC′)

To evaluate the incorporation of acetylated monolignols into the lignin of the three materials, resulting in γ-acetylated lignin side-chains, a modification of the standard DFRC method was used (Ralph and Lu, 1998). Milled lignins (5 mg) were stirred for 2 h at 50°C with propionyl bromide in propionic acid (8:92, v/v). The solvents and excess bromide were removed by rotary evaporation. The products were dissolved in dioxane/propionic acid/water (5:4:1, v/v/v), and 50 mg powdered Zn were added. The mixture was maintained for 40 min at room temperature with stirring, and transferred into a separator funnel with dichloromethane and saturated ammonium chloride. The aqueous phase was adjusted to pH < 3 by adding 3% HCl, the mixture was vigorously mixed and the organic layer separated. The water phase was extracted twice with dichloromethane. The combined dichloromethane fractions were dried over anhydrous NaSO_4_ and the filtrate was evaporated to dryness using a rotary evaporator. The residue was propionylated for 1 h in 1.1 mL of dichloromethane containing 0.2 mL of propionic anhydride and 0.2 mL pyridine. The propionylated (and naturally acetylated) lignin degradation compounds were collected after rotary evaporation of the solvents, and subsequently analyzed by GC/MS. The GC analyses were performed with a GCMS-QP2010 Ultra instrument (Shimadzu Co.) using a capillary column (DB-5HT, 30 m × 0.25 mm I.D., 0.10 μm film thickness). The oven was heated from 140°C (1 min) to 250°C at 3°C min^−1^, then ramped at 10°C min^−1^ to 300°C and held for 10 min at the final temperature. The injector was set at 250°C and the transfer line was kept at 300°C. Helium was used as the carrier gas at a rate of 1 mL min^−1^.

## Results

### Anatomy and chemical composition

**Figure 1:**
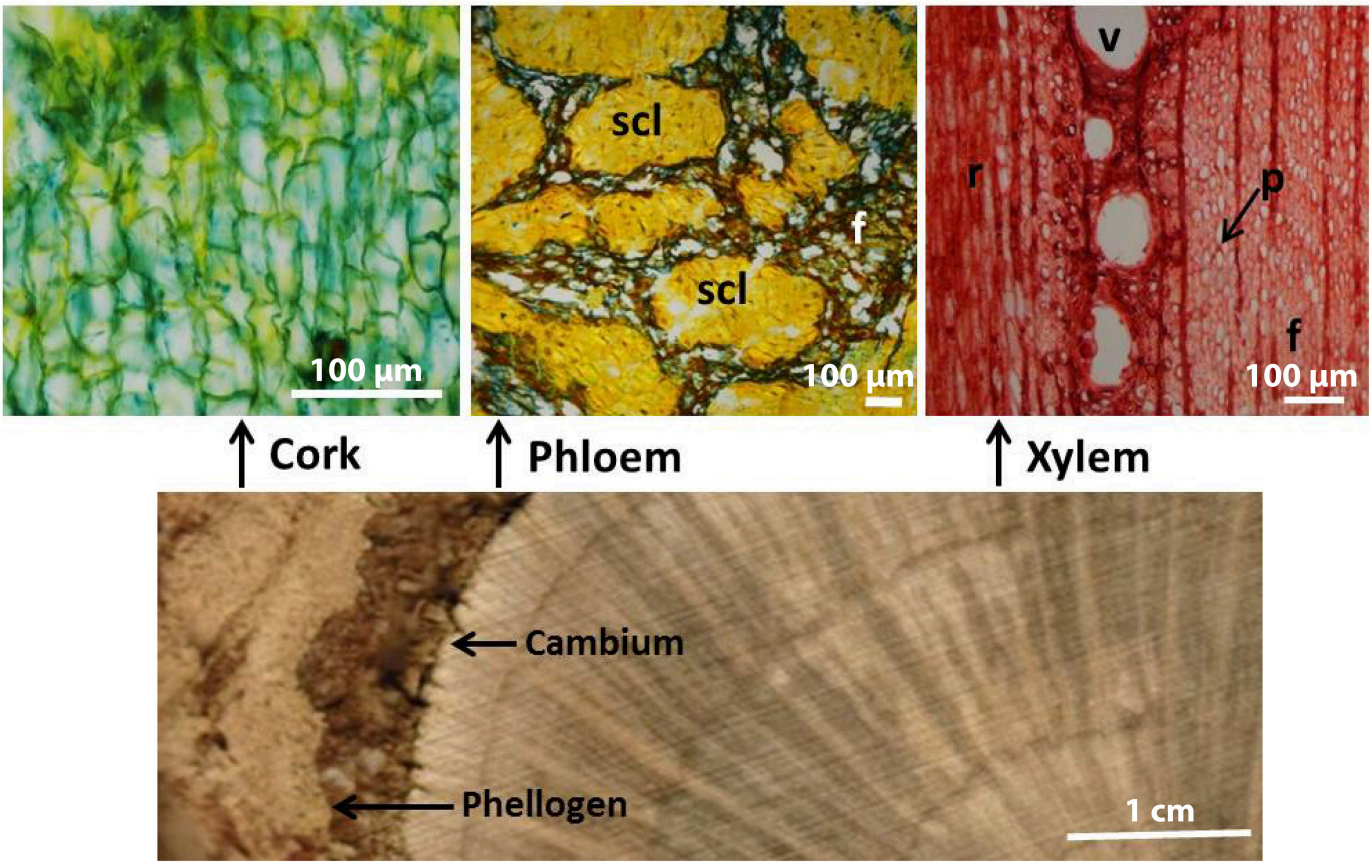
**Cross-sectional image of a *Quercus suber* stem disc presenting cork, phloem, and xylem (scale bar = 1 cm), and transverse microscopic sections of cork, phloem, and xylem tissues (scale bar = 100 μm)**. scl, clusters of sclereids; f, fibers; v, vessel; r, rays; p, parenchyma.

The location of the three tissues (cork, phloem and xylem) in the plant stem and their anatomical structure are shown in Figure 1. While cork is a homogeneous tissue of phellem cells, the phloem presents sieve elements, parenchyma, and sclerenchyma cells with sclereids, and the xylem has rays and axial parenchyma, vessels and fibers. The chemical summative composition of the xylem, phloem and cork tissues was determined in the 40–60 mesh fraction and the results are presented in Table 1. The different tissues presented great differences in composition, with cork having suberin as the major structural component. The lignin content also differed among the different tissues, accounting for 27.1% in cork, 38.4% in phloem and 23.6% in xylem.

**Table 1:** **Chemical composition of the 40–60 mesh from cork, phloem and xylem of *Quercus suber* L. Mean values of two samples**.

**(% oven dry material)**	**Cork**	**Phloem**	**Xylem**
Ash	0.7	3.1	1.5
Total extractives	10.4	6.2	8.4
Dichloromethane	4.1	0.1	0.6
Ethanol	2.9	1.9	2.8
Water	3.4	4.2	5.0
Total lignin	27.1	38.4	23.6
Klason lignin	26.2	36.0	20.6
Soluble lignin	0.9	2.4	3.0
Suberin	30.1	–	–
**MONOSACCHARIDES**			
Arabinose	2.0	0.9	0.6
Xylose	6.4	15.5	13.7
Mannose	0.4	0.1	0.6
Galactose	1.1	0.8	1.1
Glucose	9.0	16.5	28.7

### Lignin composition as determined by Py-GC/MS

**Figure 2:**
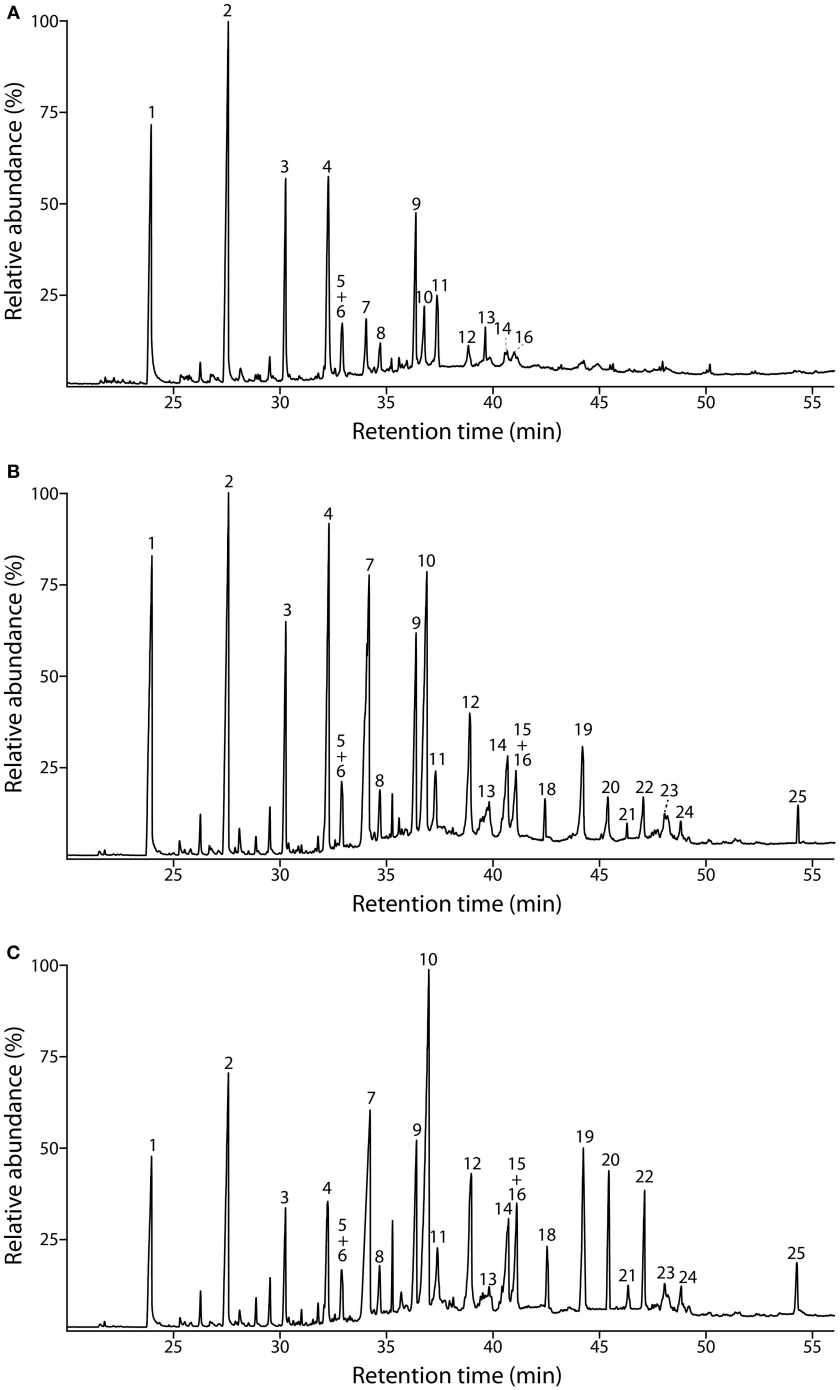
**Py-GC/MS chromatograms of the milled lignin preparations isolated from the different parts of *Q. suber* (A) cork, (B) phloem, and (C) xylem**. The identities and relative abundances of the released lignin-derived compounds are listed in Table 2.

The composition of the lignins from the different tissues was first addressed by Py-GC/MS. The pyrograms of the ML preparations isolated from cork, phloem and xylem are presented in Figure 2. The identities and relative molar abundances of the released lignin-derived phenolic compounds are listed in Table 2. The pyrograms revealed strong differences in lignin composition among the three tissues. Pyrolysis of cork lignin released predominantly phenolic compounds derived from G-lignin units, with guaiacol (compound **1**, Table 2, Figure 2), 4-methylguaiacol (**2**) and 4-vinylguaiacol (**4**) as the major compounds released, with only few amounts of S-lignin units, and presenting a strikingly low S/G ratio of 0.10. The pyrograms of the lignins isolated from phloem and xylem released more S-lignin units, particularly syringol (**7**) and 4-methylsyringol (**10**), and which were more abundant in the xylem. These differences are reflected in their higher S/G molar ratio, accounting for 0.62 in phloem lignin and 1.66 in xylem lignin.

**Table 2:** **Identities and relative molar abundances (% of identified products) of the lignin-derived compounds from pyrolysis of milled lignins of cork, phloem and xylem of *Quercus suber* L. Peak assignment from Figure 2**.

**Peak**	**Compound**	**Origin**	**Cork**	**Phloem**	**Xylem**
1	guaiacol	G	21.3	13.1	7.3
2	4-methylguaiacol	G	26.7	15.8	10.0
3	4-ethylguaiacol	G	9.7	5.6	2.8
4	4-vinylguaiacol	G	14.1	11.6	4.5
5	eugenol	G	1.3	1.0	1.4
6	4-propylguiacol	G	1.9	1.5	1.4
7	syringol	S	3.1	12.3	14.0
8	*cis*-isoeugenol	G	1.3	1.4	1.4
9	*trans*-isoeugenol	G	7.2	5.9	5.1
10	4-methylsyringol	S	2.9	8.9	15.9
11	vanillin	G	3.2	2.6	2.4
12	4-ethylsyringol	S	1.3	2.2	4.4
13	acetovanillone	G	2.4	2.3	1.3
14	4-vinylsyringol	S	1.1	3.5	3.8
15	4-propylsyringol	S	0.0	0.5	2.9
16	guaiacylacetone	G	1.7	0.9	0.0
17	4-allylsyringol	S	0.1	0.8	2.9
18	*cis*-4-propenylsyringol	S	0.2	1.0	1.6
19	*trans*-propenylsyringol	S	0.6	3.7	5.8
20	syringaldehyde	S	0.0	1.6	4.9
21	homosyringaldehyde	S	0.0	0.2	0.6
22	acetosyringone	S	0.0	1.7	2.6
23	syringylacetone	S	0.0	0.7	1.1
24	propiosyringone	S	0.0	0.5	0.8
25	*trans*-sinapaldehyde	S	0.0	0.5	1.1
S/G molar ratio			0.10	0.62	1.66

G, Guaiacyl derived units; S, Syringyl derived units. Mean values of two samples.

### Lignin structural units and inter-unit linkages analyzed by 2D-NMR

**Figure 3:**
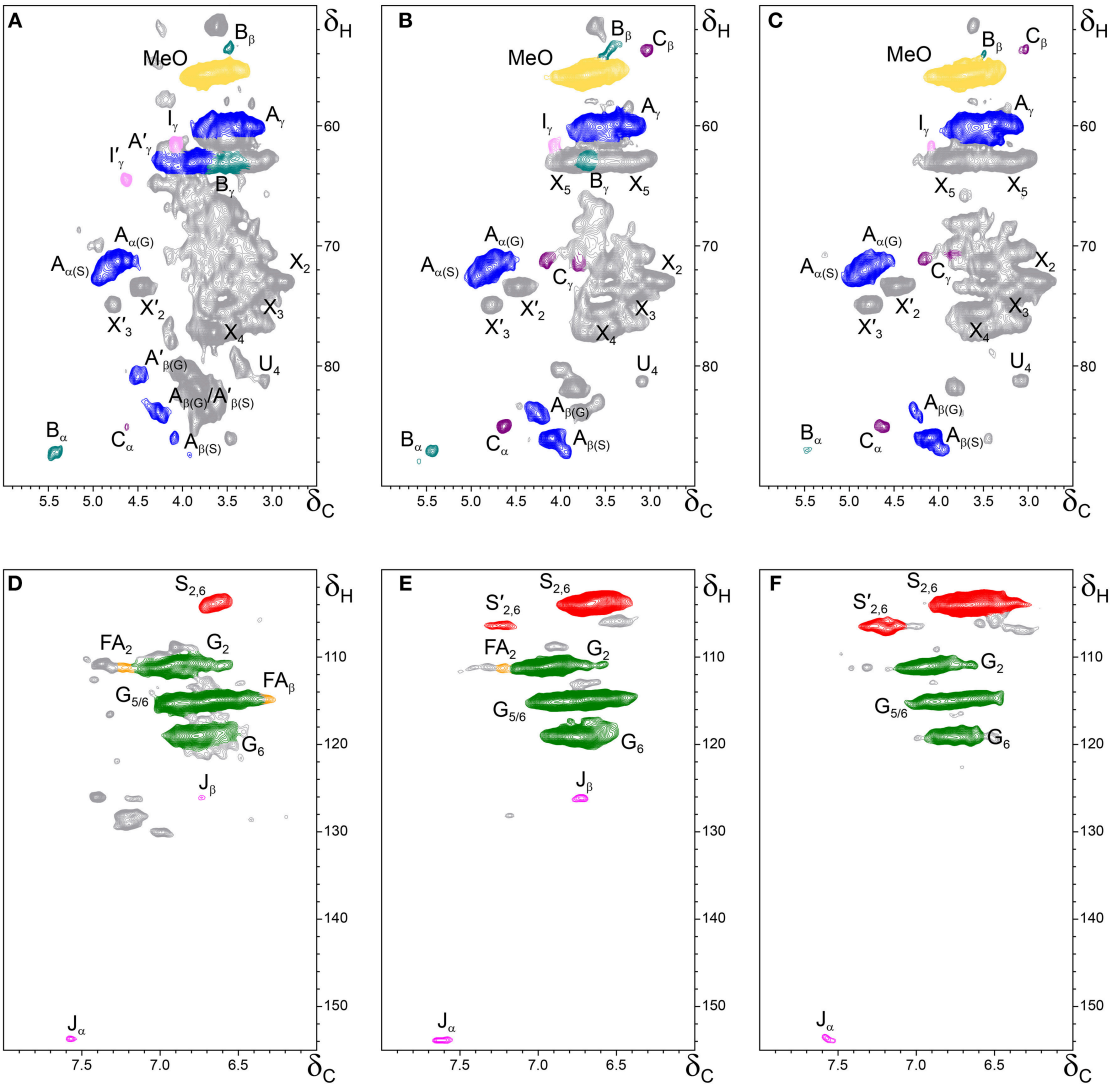
**Side-chain (δ_C_/δ_H_ 50–90/2.5–6.0) and aromatic/unsaturated (δ_C_/δ_H_ 100–155/6.0–8.0) regions in the 2D HSQC NMR spectra of the whole cell-walls from the different parts of *Q. suber* (A,D) cork, (B,E) phloem and (C,F) xylem**. The signal assignments are presented in Table 3 and the main lignin structures identified are depicted in Figure 5.

**Figure 4:**
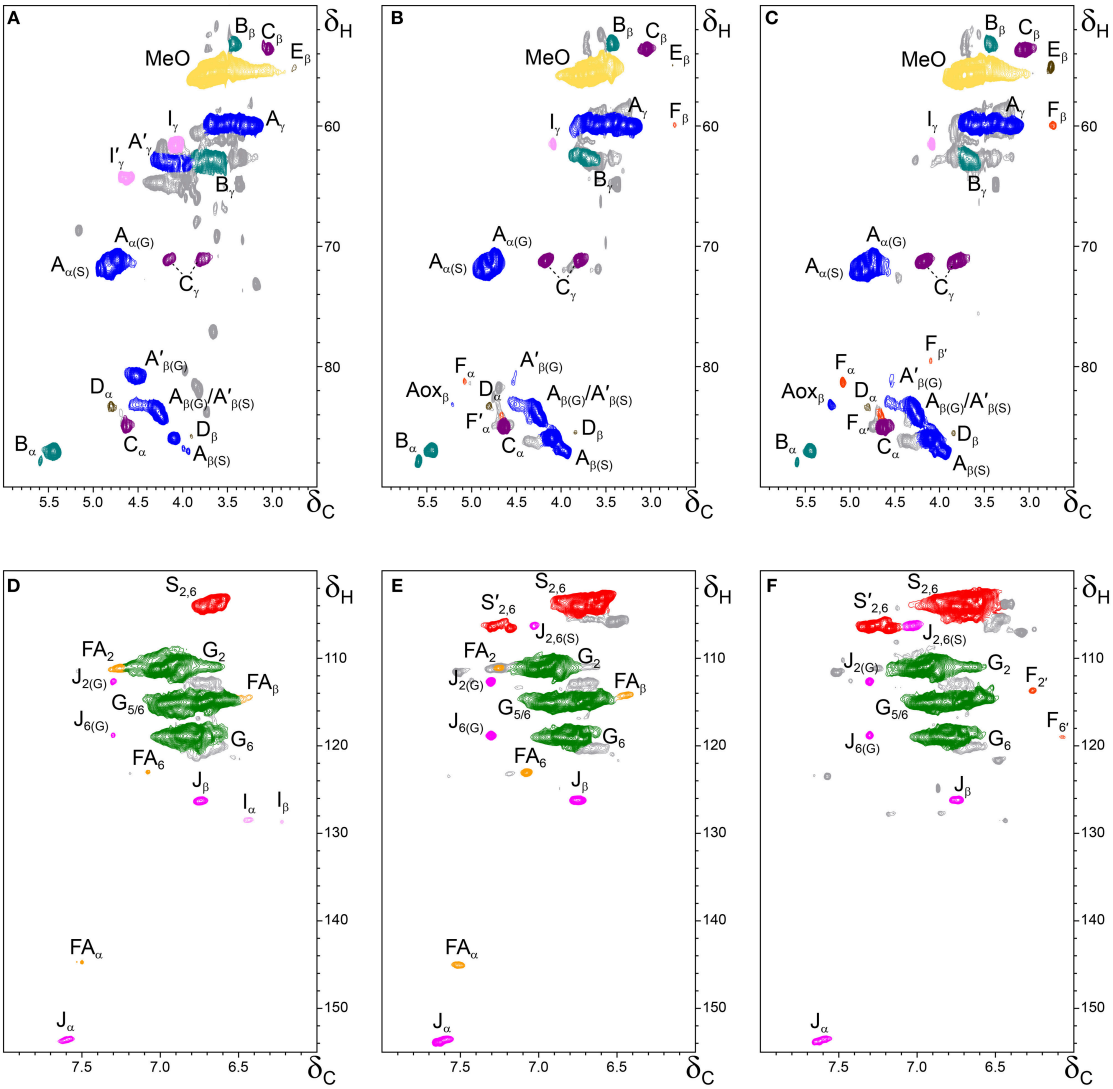
**Side-chain (δ_C_/δ_H_ 50–90/2.5–6.0) and aromatic/unsaturated (δ_C_/δ_H_ 100–155/6.0–8.0) regions in the 2D HSQC NMR spectra of the milled lignin preparations isolated from the different parts of *Q. suber* (A,D) cork, (B,E) phloem, and (C,F) xylem**. The signal assignments are presented in Table 3 and the main lignin structures identified are depicted in Figure 5.

**Figure 5:**
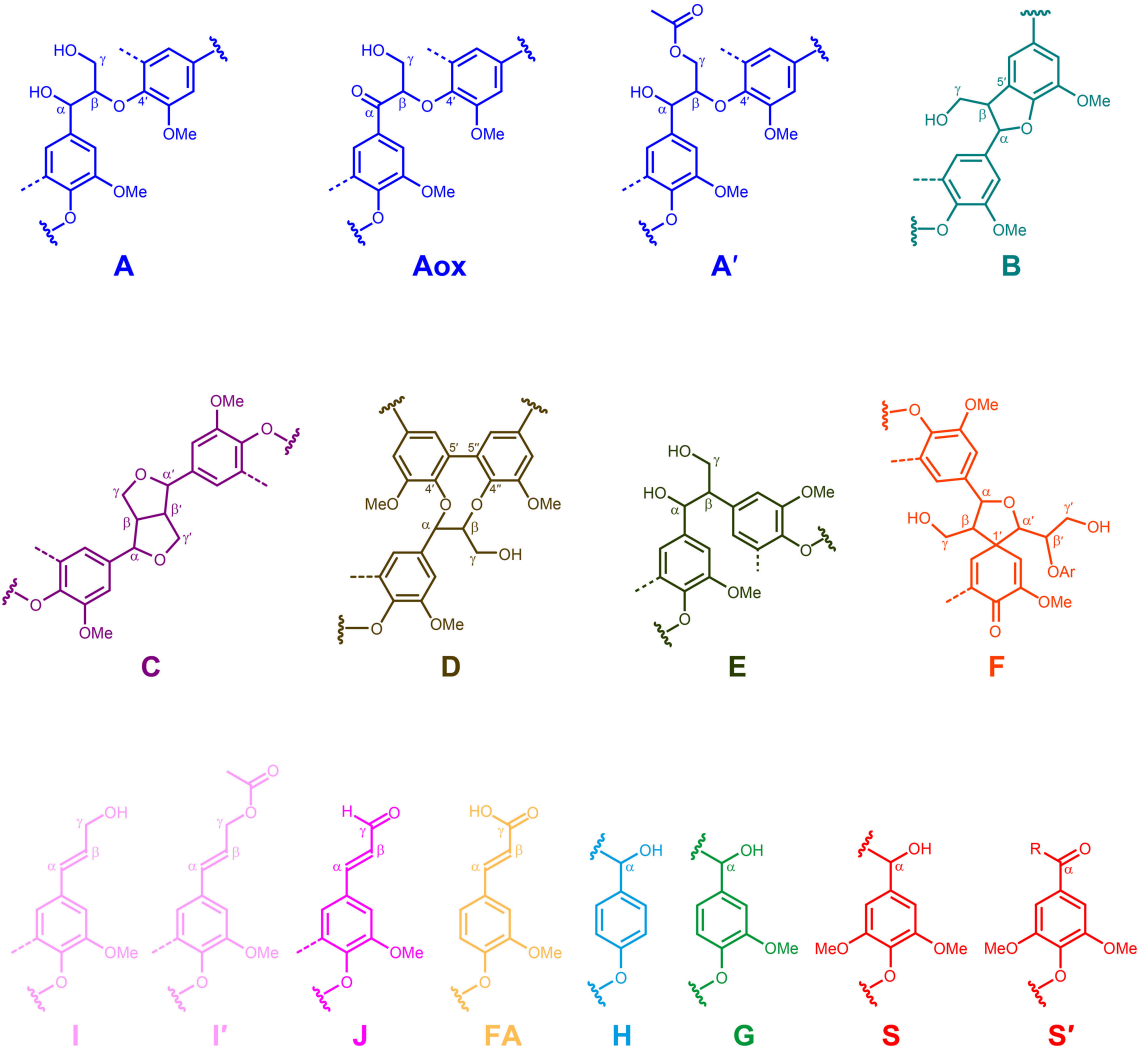
**Main structures present in the lignins from *Q. suber* cork, phloem and xylem: A, β-*O*-4′ alkyl-aryl ethers; A_ox_, α-oxidized β–*O*–4′ aryl ethers; A′, β-*O*-4′ alkyl-aryl ethers with acylated γ-OH; B, phenylcoumarans; C, resinols; D, dibenzodioxocins; E, open β–1′ structures; F spirodienones; I, cinnamyl alcohol end-groups; I′, γ-acylated cinnamyl alcohol end-groups; J, cinnamaldehyde end-groups; FA, ferulate moieties; H, *p*-hydroxyphenyl units; G, guaiacyl units; S, syringyl units; S′, oxidized syringyl units unit bearing a carbonyl group at Cα**.

The whole cell-walls of cork, phloem and xylem were analyzed by 2D HSQC NMR (Figure 3) and the spectra compared with those of their isolated lignins (Figure 4). The main lignin cross-signals assigned in the spectra are listed in Table 3, and the main lignin substructures found are represented in Figure 5. The spectra of the whole cell-walls (Figure 3) presented signals from carbohydrates, including xylan correlations in the range δ_C_/δ_H_ 60–85/2.5–5.5 (for X_2_, X_3_, X_4_, and X_5_) and signals from acetylated xylan moieties (X′_2_ and X′_3_) as well as signals from lignin, whereas the spectra of the isolated lignins (Figure 4) presented only signals from lignin. In general terms the signals of isolated lignins match those observed in the whole cell-walls, indicating that the MWL preparations used for the present work are representative of the native lignins in the cell-walls.

**Table 3:** **Assignments of the lignin ^13^C–^1^H correlation peaks in the 2D-HSQC spectra of whole cell walls and of the corresponding isolated milled lignins from cork, phloem and xylem of *Quercus suber* L**.

**Label**	**δ_C_/δ_H_**	**Assignment**
B_β_	53.1/3.43	C_β_–H_β_ in phenylcoumaran substructures (**B**)
C_β_	53.5/3.05	C_β_–H_β_ in β–β′ resinol substructures (**C**)
−OCH_3_	55.6/3.73	C−H in methoxyls
A_γ_	59.4/3.40 and 3.72	C_γ_–H_γ_ in β–*O*–4′ substructures (**A**)
D_β_	59.5/2.75	C_β_–H_β_ in in 5-5′ (dibenzodioxocin) substructures (**D**)
I_γ_	61.3/4.08	C_γ_–H_γ_ in cinnamyl alcohol end-groups (**I**)
B_γ_	62.6/3.67	C_γ_–H_γ_ in phenylcoumaran substructures (**B**)
A′_γ_	63.5/3.83 and 4.30	C_γ_–H_γ_ in γ-acylated β–*O*–4′ substructures (**A**′)
I′_γ_	64.3/4.63	C_γ_–H_γ_ in γ-acetylated cinnamyl alcohol end-groups (**I**′)
C_γ_	71.0/3.83 and 4.19	C_γ_–H_γ_ in β–β′ resinol substructures (**C**)
A_α(G)_	71.0/4.73	C_α_–H_α_ in β–*O*–4′ substructures (**A**) linked to a G-unit
A_α(S)_	71.7/4.83	C_α_–H_α_ in β–*O*–4′ substructures (**A**) linked to a G-unit
F_β′_	79.4/4.10	C_β_′–H_β_′ in spirodienone substructures (**F**)
A′_β(G)_	80.7/4.51	C_β_–H_β_ in γ-acetylated β–*O*–4′ substructures linked to a G-unit (**A**′)
F_α_	81.2/5.01	C_α_–H_α_ in spirodienone substructures (**F**)
D_α_	83.0/4.82	C_α_-H_α_ in 5-5′ (dibenzodioxocin) substructures (**D**)
F_α′_	83.6/4.68	C_α_′–H_α_′ in spirodienone substructures (**F**)
A_β(G)_	83.7/4.26	C_β_–H_β_ in β–*O*–4′ substructures (**A**) linked to a G unit
C_α_	84.7/4.64	C_α_–H_α_ in β–β′ resinol substructures (**C**)
D_β_	85.2/3.85	C_β_-H_β_ in 5-5′ (dibenzodioxocin) substructures (**D**)
A_β(S)_	85.8/4.09	C_β_–H_β_ in β–*O*–4′ substructures linked (**A**) to a S unit
B_α_	86.8/5.43	C_α_–H_α_ in phenylcoumaran substructures (**B**)
S_2,6_	103.7/6.68	C_2_–H_2_ and C_6_–H_6_ in etherified syringyl units (**S**)
J_2,6(S)_	106.2/7.02	C_2_-H_2_ and C_6_-H_6_ in sinapaldehyde end-groups (**J**)
S′_2,6_	106.3/7.32 and 7.20	C_2_-H_2_ and C_6_-H_6_ in C_α_-oxidized syringyl units (**S**′)
G_2_	110.8/6.96	C_2_–H_2_ in guaiacyl units (**G**)
FA_2_	111.1/7.25	C_2_-H_2_ in ferulates (**FA**)
J_2(G)_	112.5/7.30	C_2_–H_2_ in conyferaldehyde end-groups (**J**)
F_2′(S)_	113.5/6.25	C_2_′–H_2_′ in spirodienone substructures (**F**)
FA_β_	113.5/6.27	C_β_–H_β_ in ferulates (**FA**)
G_5_/G_6_	115.0/6.74	C_5_–H_5_ and C_6_–H_6_ in guaiacyl units (**G**)
G_6_	118.7/6.77	C_5_–H_5_ inguaiacyl units (**G**)
J_6(G)_	118.8/7.30	C_6_–H_6_ in conyferaldehyde end-groups (**J**)
F_6′(S)_	118.9/6.06	C_6_′–H_6_′ in spirodienone substructures (**F**)
FA_6_	123.3/7.10	C_6_–H_6_ in ferulate (**FA**)
J_β_	126.3/6.76	C_β_–H_β_ in cinnamyl aldehyde end-groups (**J**)
H_2,6_	128.0/7.23	C_2,6_–H_2,6_ in *p*-hydroxyphenyl units (**H**)
FA_α_	144.4/7.41	C_α_–H_α_ in ferulates (**FA**)
J_α_	153.4/7.61	C_α_–H_α_ in cinnamyl aldehyde end-groups (**J**)

The aliphatic-oxygenated region of the spectra (around δ_C_/δ_H_ 50−90/2.5−6.0, Figures 3A–C, 4A–C) gives information on the inter-unit linkages in lignin. In this region, cross-signals from methoxyl groups and from β−*O*−4′ alkyl-aryl ethers (structure **A**) are predominant in all samples, although differing in their intensities. Signals from other lignin substructures were also detected in the HSQC spectra, although with lower intensities, including signals from phenylcoumarans (**B**), resinols (**C**), dibenzodioxocins (**D**), open β-1 structures (**E**), spirodienones (**F**), and cinnamyl alcohol end-groups (**I**). This region of the spectrum can also provide information on the acylation degree in lignin. The HSQC spectrum of cork lignin (Figure 4A) clearly showed the occurrence of intense signals in the range from δ_C_/δ_H_ 63.5/3.83-4.30 and at 64.3/4.63, that correspond to the C_γ_-H_γ_ correlations of γ-acylated β−*O*−4′ alkyl-aryl ethers units (structures **A**′**)** and γ-acylated cinnamyl alcohol end-groups (**I**′). This indicates that cork lignin is partially acylated at the γ-position of the lignin side-chain. The estimation of the γ-acylation was accomplished by integration of the signals corresponding to the C_γ_-H_γ_ correlations of the γ-hydroxylated (**A**) vs. γ-acylated (**A**′) structures, and indicated a 48% acylation degree of the lignin side-chains in cork. The HSQC spectra of the whole cell-walls of cork (Figures 3A,D) and their isolated ML (Figures 4A,D) showed the presence of a signal at δ_C_/δ_H_ 80.7/4.51 characteristic for the C_β_-H_β_ correlations of γ-acylated β−*O*−4′ substructures (**A**′) linked to G-units (del Río et al., 2012a,b, 2015), indicating a significant degree of γ-acylation of G-lignin units in the cork lignin. Signals from acylated lignin were not observed in the spectra of phloem (Figures 4B,E) and xylem (Figures 4C,F), indicating that these lignins are not acylated, or only to a very low extent. The aromatic region of the spectra (around δ_C_/δ_H_ 100−155/6.0−8.0, Figures 3D–F, **4D–F**) shows the signals from the aromatic rings and unsaturated side-chains of the different H-, G-, and S-lignin units, as well as from ferulates (structure **FA**). Signals from cinnamyl alcohol (**I**) and cinnamyl aldehyde end-groups (**J**) are also present in this region of the spectra. The content in cinnamaldehyde end-groups was estimated after comparing the intensities of C_β_-H_β_ correlations in cinnamyl alcohols (**I**) and aldehydes (**J**).

The relative abundances of the main lignin inter-unit linkages and end-groups, as well as the percentage of γ-acylation, the molar abundances of the different lignin units (H, G, and S) and ferulates, and the S/G ratios of the lignins in the cork, phloem and xylem of *Q. suber*, estimated from volume integration of contours in the HSQC spectra, are shown in Table 4. Important differences were observed in the composition and structure of the lignins from the three tissues. The lignin from cork is enriched in G-units, with a H:G:S molar composition of 2:85:13, whereas the lignin from phloem has less G-units (H:G:S of 1:58:41), and the lignin from xylem is enriched in S-units (H:G:S of 1:45:55). The S/G ratios estimated by 2D-NMR were 0.1 in cork, 0.7 in phloem, and 1.6 and 1.2 in xylem (respectively in cell walls and isolated lignin). These values match quite closely those determined by Py-GC/MS, as reported above. These compositional differences were also reflected in the relative abundances of the different inter-unit linkages. β−*O*−4′ alkyl-aryl ethers are the most predominant linkages in the three lignins, but their relative abundances increase from 68% in cork, to 71% in phloem and to 77% in xylem, consistent with the enrichment in S-lignin units. Cork lignin is enriched in condensed linkages such as phenylcoumarans (20%), dibenzodioxocins (5%) and resinols (4%). On the opposite, phloem and xylem present a lignin with less phenylcoumarans (13 and 9% respectively), dibenzodioxocins (2 and 1%) but more resinols (7 and 8%) and a small amount of open β−1 structures (2 and 1%). Signals from cinnamyl alcohol (**I**) and cinnamaldehyde end-groups (**J**) were also observed, particularly in cork lignin, which is enriched in end-groups (5% of acylated cinnamyl alcohol, 8% of cinnamyl alcohol and 11% of cinnamaldehyde). These end-groups are also present, although in lower amounts, in the lignins from phloem and xylem. Finally, Table 4 shows the high extent of γ-acylation of the cork lignin (48%) that contrasts with the absence of lignin acylation in phloem and xylem tissues.

**Table 4:** **Structural characteristics (lignin inter-unit linkages, end-groups, γ-acylation, aromatic units and S/G ratio, and ferulate content) from Integration of ^13^C-^1^H correlation peaks in the HSQC Spectra of the whole cell-walls (CW) and isolated milled lignins (ML) of xylem, phloem, and cork from *Quercus suber* L**.

	**Cork CW**	**Cork ML**	**Phloem CW**	**Phloem ML**	**Xylem CW**	**Xylem ML**
**LIGNIN INTER-UNIT LINKAGES (%)**						
β–*O*–4′ aryl ethers (**A/A**′)	−	68	−	71	−	77
α-oxidized β–*O*–4′ aryl ethers (**A_ox_**)	−	0	−	2	−	2
Phenylcoumarans (**B**)	−	20	−	13	−	9
Resinols (**C**)	−	4	−	7	−	8
Dibenzodioxocins (**D**)	−	5	−	2	−	1
Open β-1 (**E**)	−	0	−	2	−	1
Spirodienones (**F**)	−	3	−	3	−	2
**LIGNIN END-GROUPS^a^**						
Cinnamyl alcohol end-groups (**I**)	−	8	−	2	−	1
γ-acylated cinnamyl alcohol end-groups (**I**′)	−	5	−	0	−	0
Cinnamaldehyde end-groups (**J**)	−	11	−	7	−	4
Lignin side-chain γ-acylation (%)	−	48	−	0	−	0
**LIGNIN AROMATIC UNITS^b^**						
H (%)	8^*^	2	1	1	0	1
G (%)	84	85	59	58	39	45
S (%)	8	13	40	41	61	55
S/G ratio	0.1	0.1	0.7	0.7	1.6	1.2
Ferulates (%)^c^	5	6	6	5	0	0

a Expressed as a fraction of the total lignin inter-unit linkage types **A–F**.

b Molar percentages (H + G + S = 100).

c Ferulate molar content as percentages of total lignin content (H + G + S).

* Content of H-units are overestimated due to the occurrence of signals from proteins.

### Evaluation of acylation groups by DFRC′ analysis

As mentioned before, cork lignin is partially acylated at the γ-position of the side-chain (48% of the units), while the lignins in phloem and xylem were not acylated. However, the nature of the acylating group could not be assessed by HSQC. Information regarding the nature of the acylation of the γ-OH was obtained from DFRC, a degradation method that cleaves α- and β-ether linkages in the lignin polymer leaving γ-esters intact and therefore is appropriate for analysis of γ-acylated lignins (Lu and Ralph, 1997a,b, 1998). The method was slightly modified (so called DFRC′) by replacing acetylating reagents with propionylating ones in order to evaluate the presence of acetate groups originally acylating the lignin γ-OH (Ralph and Lu, 1998; del Río et al., 2007b).

**Figure 6:**
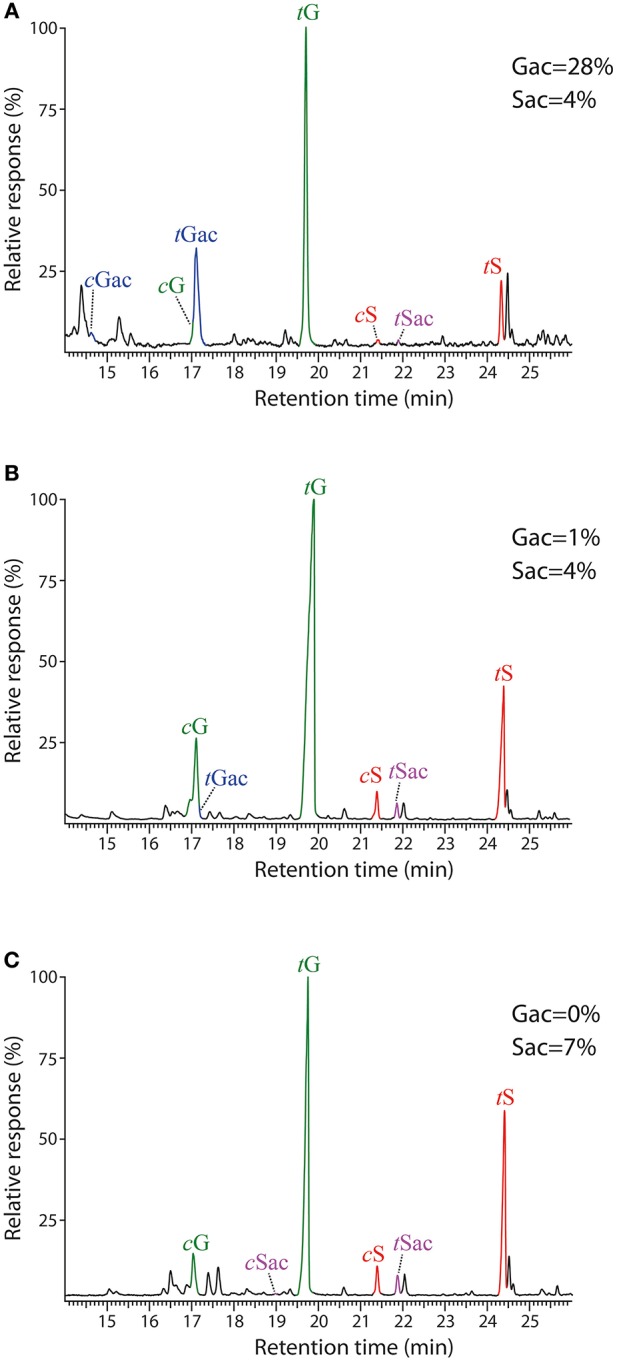
**Chromatograms of the DFRC′ degradation products from the milled lignin preparations isolated from the different parts of *Q. suber* (A) cork, (B) phloem, and (C) xylem**. *c*G, *t*G, *c*S, and *t*S are the normal *cis*- and *trans*-coniferyl (guaiacyl) and sinapyl (syringyl) alcohol monomers (as their dipropionylated derivatives). *c*G_ac_, *t*G_ac_, *c*S_ac_ and *t*S_ac_ are the natively γ-acetylated *cis*- and *trans*-coniferyl (guaiacyl) and sinapyl (syringyl) alcohol monomers (as their phenol propionylated derivatives).

The GC-MS chromatograms of the DFRC′ degradation products of the lignins isolated from cork, phloem and xylem are presented in Figure 6. The compounds released were the *cis-* and *trans-* isomers of guaiacyl (*c*G, *t*G) and syringyl (*c*S, *t*S) lignin monomers (as their propionylated derivatives) arising from normal γ-OH units in lignin. In addition, the presence of originally γ-acetylated guaiacyl (cG_ac_ and tG_ac_) and syringyl lignin units (*c*S_ac_ and *t*S_ac_) were also detected in the chromatograms, confirming that acetylation at the γ-OH of the side-chain occurred in these lignins, being particularly abundant in cork lignin, and, to a much lower extent, also in phloem and xylem lignins. In cork lignin, acetylation occurred predominantly over the guaiacyl units (28% of the total G units are acetylated), as already advanced by 2D-NMR, whereas only minor amounts of syringyl units were acetylated (4% of the S-units). In contrast, the lignins from phloem and xylem were acetylated only to a very minor extent (not observed by 2D-NMR) and predominantly over S-units.

## Discussion

The chemical composition of cork and xylem (Table 1) is in general agreement with the values reported in the literature for *Q. suber* cork (Pereira, 1988, 2013; Jové et al., 2011) and wood (Leal et al., 2005). However, there are no studies reporting the chemical composition of *Q. suber* phloem, although a previous study comparing the chemical composition of reproduction cork and its phloemic outside layer (i.e. the outer phloem external to the formation of the traumatic phellogen) reported a similar compositional difference, with a higher lignin content for the outer phloem (32.5%) than for the cork (23.0%) (Pereira, 1987). For other species in which phloem and cork have been analyzed (e.g., *P. menziesii* and *Q. cerris*), the lignin content was also considerably higher in phloem than in cork (Sen et al., 2010; Ferreira et al., 2014). Our data is therefore in agreement with previous reports and indicate a higher lignin content in phloem with respect to cork and xylem tissues. The high lignification of phloem is associated to the conspicuous presence of thick-walled and heavily lignified fibers and sclereids, as Figure 1 clearly exemplifies.

The composition of the lignin in the three tissues presented great differences. Whereas cork lignin is enriched in G-units (S/G of 0.1), the lignin from phloem has less G- and more S-units (S/G of 0.7) and the lignin from xylem is enriched in S-units (S/G of 1.2). The H:G:S composition of the three lignins therefore indicates a continuous decrease in the content of H- and G-units and an enrichment in S-units from the cork to phloem to xylem. The strong predominance of G-units present in the lignin from *Q. suber* cork and in the lignin from the corks of other species (such as *B. pendula* and *Q. cerris*) has already been reported (Marques et al., 1994, 1996, 2006; Marques and Pereira, 2013). There is no report in the bibliography on the monomeric composition of phloem-only lignin. However the S/G ratio for the bark of *T. grandis* that is mostly constituted by phloem i.e., with a very small proportion of cork, presented a very similar value of 0.8 (Lourenço et al., 2015). As regards to the xylem, there is no data for *Q. suber*, but for *Q. robur* the S/G ratio was found 1.9 (Karami et al., 2013).

The results obtained are a striking confirmation that the monomeric composition of lignin is different depending on cell type and tissue (Barros et al., 2015) and that the lignin distribution is differentially regulated depending on cell types (Nakashima et al., 2008; Saito et al., 2012). The monomeric composition of lignin is largely determinant to the inter-monomeric linkages and polymer structure, which may have implications regarding functional requirements of strength and protection; for instance, S-lignin is less condensed i.e., fewer C-C interunit bonds than a G-lignin.

Tracheary elements require a reinforcement of their lateral cell walls in order to be able to withstand the negative pressure of sap ascent; therefore, they are mainly composed of G-units; while fibers and sclereids, that provide general mechanical strength, have mostly S-units (Terashima and Fukushima, 1989; Higuchi, 1990). This may explain the differing monomeric composition of xylem, phloem and cork in *Q. suber*: the xylem has a large proportion of fibers (Sousa et al., 2009), and phloem a large content of sclereids (Figure 1) and both have a higher content of S-units, while the cork cells are the external protective layer of the plant and have a G-lignin. The distribution of the different lignin inter-unit linkages (Table 4) is closely related to the proportion of the different lignin monomers; therefore, the cork lignin, due to the predominance of G-units, presents less β-*O*-4 aryl ethers (**A/A**′) and more condensed structures such as phenylcoumarans (**B**) and dibenzodioxocins (**D**). This is consistent with the protective function of cork toward external stresses. In fact, protective barriers such as the Casparian strips also have a lignin with more H- and G-units than S-units (Barros et al., 2015).

Ferulates were present in important amounts (ca. 5%) in the lignin from phloem and cork but were completely absent in the lignin from xylem. Ferulic acid units make the link between lignin and carbohydrates (Ralph and Landucci, 2010) and are present in suberized cell walls, chemically bridging suberin and lignin as shown recently for *Q. suber* cork (Marques et al., 2015).

The timing of lignification is also a potential explanation for the structural difference of cork lignin in relation to wood and phloem lignins. Terashima et al. (1986) reported that the deposition of lignin units in the cell wall is sequential, *p*-coumaryl alcohol (H-units) are deposited first, followed by coniferyl alcohol (G-units) and then by sinapyl alcohol (S-units). Studies by microautoradiography and microspectroscopy also showed that the incorporation of G-units continues throughout the early to late stages of xylem differentiation, while the S-units are deposited mainly during the middle and late stages (Terashima et al., 1986; Fukushima and Terashima, 1991; Rencoret et al., 2011). A study conducted in the cambial zone of poplar during a growth season showed that the cells had more G-units in the early stages of differentiation, and also that phloem cells had more G-units comparatively to wood cells (Christiernin, 2006). Therefore, a more rapid lignin deposition in the cell wall will lead to more G-units and a more condensed structure. In the case of cambium-derived cells, such as tracheary elements and sclerenchyma cells, lignification occurs in the final stages of cell differentiation during wall thickening and proceeds in sequential phases after deposition of polysaccharides (Donaldson, 2001). This explains why different cells have different lignin composition; since the vessel walls lignify earlier than fiber walls, they contain mainly G-units while fibers contain less G- and more S-units. In the case of cork, the process of cell wall thickening with suberin deposition is very quick in the cells neighboring the phellogen mother-cell and the process spans only to a few cells (Teixeira and Pereira, 2009). With ^14^C-labeling of young cork oaks, it was found that suberin was a highly effective sink for the carbon assimilated with a fast synthesis (Aguado et al., 2012). The anatomical features of the cork cells e.g., leading to the cell wall corrugations shown by the radial cell walls are also indicative of a very rapid lignification process (Pereira, 2015). This explains the enrichment in G-lignin units of cork cells.

On the other hand, our data indicate that the lignin from cork was highly acylated at the γ-OH with acetate groups, and that acetylation occurred predominantly over the G-units. This finding is quite remarkable since in most plants γ-acetylation occurs predominantly on S-units, where sinapyl acetate acts as a real monolignols and is involved in coupling and cross-coupling reactions during lignification (Ralph, 1996; Lu and Ralph, 2002; del Río et al., 2007b, 2008). This fact seems to indicate that coniferyl acetate also acts as a real monolignol in the biosynthesis of cork lignin and points to the occurrence of the corresponding acetyl transferases with a higher affinity toward coniferyl alcohol than toward sinapyl alcohol. Acylation could not be observed in the lignins from phloem and xylem by 2D-NMR, but DFRC′ analyses indicated that these lignins are also acetylated, although at a low level, and preferentially over S-units, as it has been reported in other plants (Ralph, 1996; Lu and Ralph, 2002; del Río et al., 2007b, 2008). The role of the high extent of lignin acetylation of cork lignin, compared to the minor acetylation degree of the lignins in phloem and xylem, is not yet known. However, since the resultant acetylated lignin is more hydrophobic than normal lignin, lignin acetylation will increase the hydrophobicity of the cork tissues thus helping to reduce water loss in the plant.

The reasons underlying the differences that were found in the lignin composition and structure in the cells produced by the cambium (xylem and phloem) and in the cells produced by the phellogen (cork) may only be speculatively discussed. This is certainly a subject where more focused studies have to be made in order to understand the compositional differences of lignin in the different tissues and cells. In any case, it is apparent that the mechanism of lignin biosynthesis confers the plant a high flexibility to produce different types of lignins for different tissues.

## Conclusions

This study reports the differences in composition and structure of the lignins from different tissues—cork (phellem), phloem and xylem (wood)—of *Q. suber*. The whole cell walls and their isolated milled lignins were thoroughly characterized by using different analytical methodologies (Py-GC/MS, NMR and DFRC′). The data revealed important differences in the composition and structure of the lignins among the three tissues. Cork lignin was predominantly a G-lignin (H:G:S molar ratio of 2:85:13), enriched in condensed structures such as phenylcoumarans (20%) and dibenzodioxocins (5%). In contrast, phloem has less G- and more S-units (H:G:S molar ratio of 1:58:41) and xylem has a prevalence of S-units (H:G:S molar ratio of 1:45:55), both with a predominance of alkyl-aryl ether linkages (71 and 77% of β–*O*–4′ linkages). The data also indicated that the cork lignin was extensively acetylated at the γ-OH, and mainly over G-units, contrasting with phloem and xylem lignins that presented low levels of acetylation, and predominantly over S-units. Therefore, it can be assumed that coniferyl acetate acts as a monolignol in the biosynthesis of cork lignin. These results clearly show that the lignin from cells produced by the cambium (xylem and phloem) is quite different from the lignin from cells produced by the phellogen (cork). These results points out that the differences in lignin structure and monomeric composition in lignocellulosic materials may derive from differences in cell type, proportion and in lignification kinetics, including secondary wall deposition rate.

## Author contributions

AL, JR contributed equally to the experimental part of this work: they isolated the lignins and made their characterization by Py-GC/MS, DFRC′ and 2D-NMR; CC prepared the raw material and made the chemical analysis with the technical assistance of JG that helped in the interpretation of the results; AG, JCR provided scientific assistance during lignin isolation and characterization, and contributed to the interpretation of the results; AL drafted the article; HP conceived the project and together with JCR revised and complemented the writing of the article. All authors read and approved the manuscript.

## Funding

The first author was funded by FCT through a post-doctoral grant (SFRH/BPD/95385/2013). The research was financed by the Portuguese Science Foundation (FCT) through the base funding to the Forest Research Center (CEF) under UID/AGR/00239/2013. This study has also been partially funded by the Spanish projects AGL2011-25379, AGL2014-53730-R, and CTQ2014-60764-JIN (co-financed by FEDER funds), the CSIC project 2014-40E-097 and the EU-project INDOX (KBBE-2013-7-613549).

### Conflict of interest statement

The authors declare that the research was conducted in the absence of any commercial or financial relationships that could be construed as a potential conflict of interest.
